# Association of Serum Autotaxin Levels with Liver Fibrosis in Patients with Chronic Hepatitis C

**DOI:** 10.1038/srep46705

**Published:** 2017-04-20

**Authors:** Tomoo Yamazaki, Satoru Joshita, Takeji Umemura, Yoko Usami, Ayumi Sugiura, Naoyuki Fujimori, Soichiro Shibata, Yuki Ichikawa, Michiharu Komatsu, Akihiro Matsumoto, Koji Igarashi, Eiji Tanaka

**Affiliations:** 1 Department of Medicine, Division of Hepatology and Gastroenterology, Shinshu University School of Medicine, Matsumoto, Japan; 2 Department of Laboratory Medicine, Shinshu University Hospital, Matsumoto, Japan; 3 Bioscience Division, TOSOH Corporation, Kanagawa, Japan

## Abstract

Metabolized by liver sinusoidal endothelial cells, autotaxin (ATX) is a secreted enzyme considered to be associated with liver damage. We sought to clarify the diagnostic ability of ATX for liver fibrosis in 593 biopsy-confirmed hepatitis C virus (HCV)-infected patients. The diagnostic accuracy of ATX was compared with clinical parameters and the established fibrosis biomarkers Wisteria floribunda agglutinin-positive Mac-2-binding protein, FIB-4 index, AST-to-platelet ratio, and Forn’s index. Median ATX levels were consistently higher in female controls and patients than in their male counterparts (P < 0.01). Serum ATX concentration increased significantly according to liver fibrosis stage in overall and both genders (P < 0.001). The cutoff values of ATX for prediction of fibrosis stages ≥F1, ≥F2, ≥F3, and F4 were 0.8, 1.1, 1.3, and 1.7 mg/L, respectively, in male patients and 0.9, 1.7, 1.8, and 2.0 mg/L, respectively, in female patients. The area under the receiver operating characteristic curve for ATX to diagnose fibrosis of ≥F2 (0.861) in male patients was superior to those of FIB-4 index and Forn’s index (P < 0.001), while that in female patients (0.801) was comparable with those of the other markers. ATX therefore represents a novel non-invasive biomarker for liver fibrosis in HCV-infected patients.

Persistent infection with HCV causes chronic hepatitis and eventually leads to liver cirrhosis and/or hepatocellular carcinoma (HCC)^1^. Although direct-acting antiviral agent (DAA) therapies have achieved high rates of a sustained virological response, chronic HCV infection remains a pressing health problem^2^,^3^,^4^,^5^,^6^. Disease progression to cirrhosis, hepatic failure, and complicating HCC, as well as esophageal and gastric varices, have been shown to positively correlate with histological involvement of the liver^7^. Thus, assessment of liver fibrosis stage in patients with chronic hepatitis C is deemed crucial in the decision of surveillance interval and therapeutic intervention^8^. Liver biopsy is the standard and most accurate examination method to evaluate liver fibrosis stage, but is invasive, prone to sampling error, and carries the risk of rare, but serious, complications^9^,^10^. Non-invasive biomarkers of disease progression are therefore needed. FIB-4 index^11^, aspartate aminotransferase (AST)-to-platelet ratio (APRI)^12^, and Forn’s index^13^ have all been established as non-invasive liver fibrosis markers and are useful in the clinical setting. Recently, *Wisteria floribunda* agglutinin-positive Mac-2-binding protein (WFA^+^-M2BP) was also discovered as a novel, noninvasive, serum glyco-marker for liver fibrosis in glycoproteomic biomarker screening studies^14^,^15^. This method has been validated for other liver diseases as well^16^,^17^.

Autotaxin (ATX) encoded by the *ectonucleotide pyrophosphatase*/*phosphodiesterase family member 2 (ENPP 2*) gene is a secreted enzyme originally discovered in conditioned medium from A2058 human melanoma cell cultures^18^. ATX has important functions in converting lysophosphatidylcholine to lysophosphatidic acid (1- or 2-acyl-lysophosphatidic acid; LPA)^19^ involved in such physiological roles as cell migration, neurogenesis, angiogenesis, smooth-muscle contraction, platelet aggregation, and wound healing^20^,^21^. LPA also stimulates the proliferation and contractility of hepatic stellate cells. ATX was recently implicated in cancer metastasis and invasion as an autocrine motility factor^22^. Physiologically, the enzyme exists in the serum and is metabolized by liver sinusoidal endothelial cells. ATX metabolism is therefore considered to be reduced by liver fibrosis, leading to the elevation of serum values. These findings indicate that ATX may be directly related to liver fibrosis^23^,^24^,^25^. Although ATX levels reportedly vary significantly between genders in healthy subjects^26^, they nonetheless represent a novel serum marker of liver fibrosis^27^. However, the clinical features of ATX are inconclusive due to small cohort sizes.

This study aimed to clarify the clinical characteristics of ATX in patients chronically infected with HCV based on histological assessment evidence. We also compared the fibrosis predictive ability of ATX with that of other serum liver fibrosis markers.

## Results

### Baseline clinical characteristics

The baseline clinical characteristics of our cohort are summarized in Table 1. Of the 593 patients enrolled, 292 (49.2%) were male. Median age was 58 years. The vast majority of patients were seen for histological assessment prior to antiviral treatment with interferon-based therapy or DAAs. Fibrosis stage was F0 for 8 cases (1.3%), F1 for 284 cases (47.9%), F2 for 144 cases (24.3%), F3 for 103 cases (17.4%), and F4 for 54 cases (9.1%). Comparisons of baseline clinical characteristics between genders revealed several significant differences (Table 1), although the distributions of fibrosis stage and activity grade were comparable (*P* = 0.888 and *P* = 0.171, respectively).

### ATX levels in controls and patients and between genders

**Figure 1:**
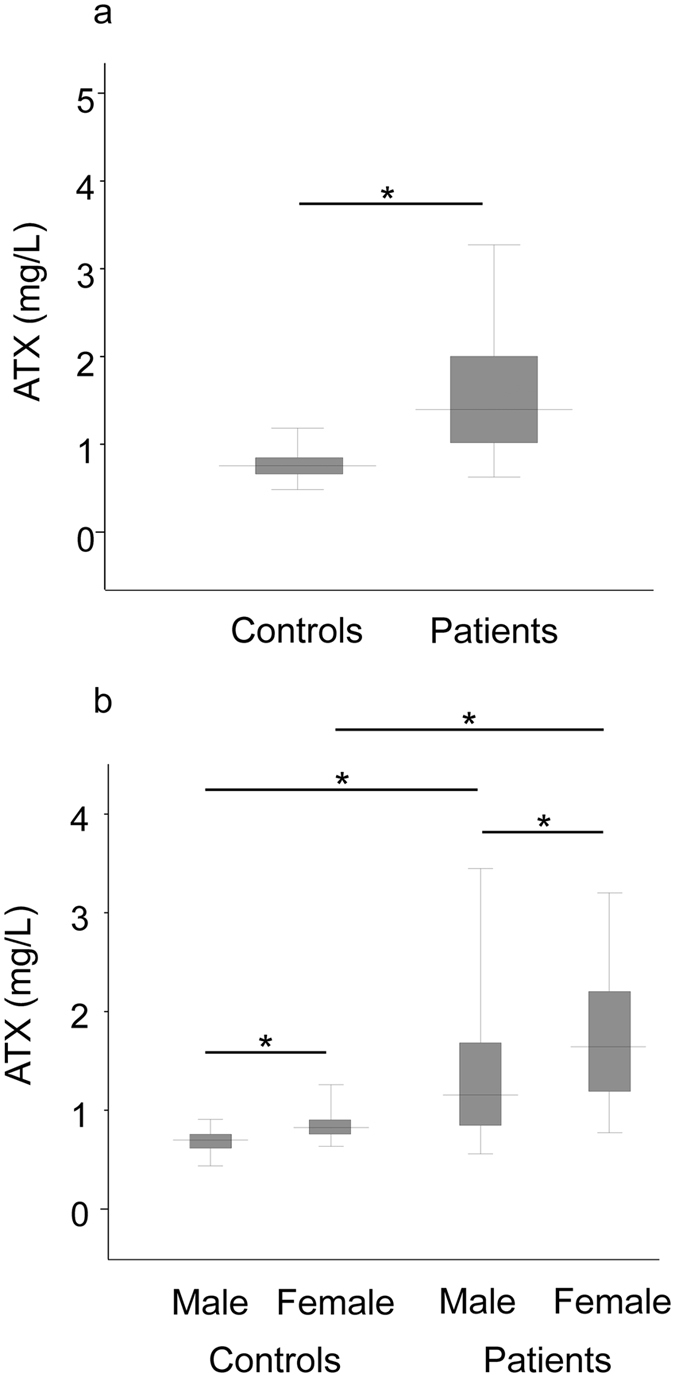
Comparison of autotaxin (ATX) levels between controls and patients with chronic hepatitis C (**a**) and according to gender (**b**). The top and bottom of each box represent the first and third quartiles, respectively. The lines across the boxes indicate median values. *P < 0.001.

Median ATX levels were significantly higher in patients than in healthy controls (1.40 vs. 0.76 mg/L; *P* < 0.001) (Fig. 1a). This relationship persisted when divided by gender (male: 1.16 vs. 0.70 mg/L; *P* < 0.001, female: 1.64 vs. 0.82 mg/L; *P* < 0.001) (Fig. 1b). In comparisons between genders, median ATX levels were significantly higher in women than in men both among healthy controls (0.82 vs. 0.70 mg/L; *P* < 0.001) as well as in patients with chronic hepatitis C (1.64 vs. 1.16 mg/L; *P* < 0.001) (Fig. 1b). Interestingly, FIB-4 index (*P* = 0.037) and Forn’s index (*P* = 0.011) were also higher in female patients, while WFA^+^-M2BP levels and APRI were comparable between genders (Table 1).

### Correlation between ATX and liver fibrosis stage

**Figure 2:**
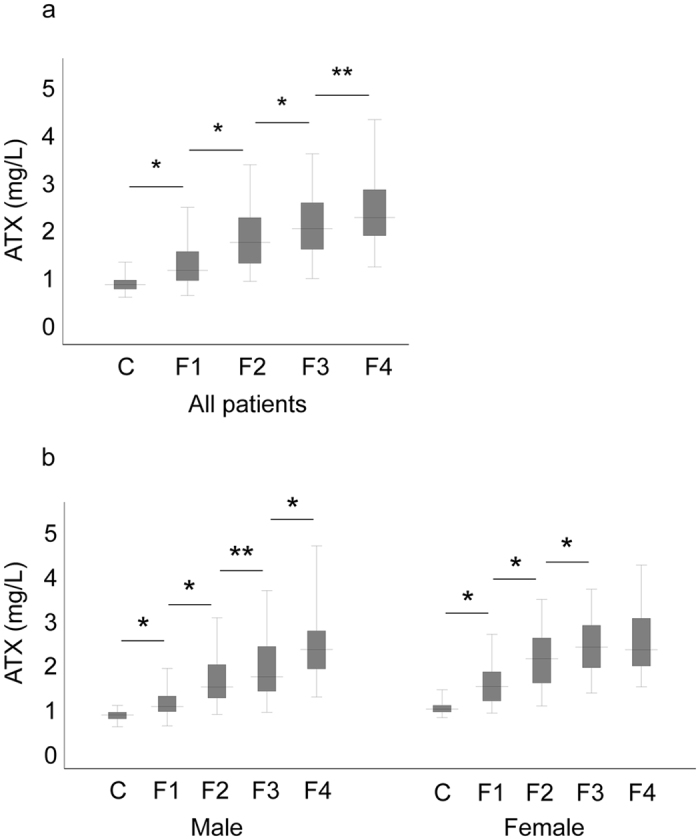
Correlation between serum autotaxin (ATX) and liver fibrosis stage in all patients (**a**) and by gender (**b**). The top and bottom of each box represent the first and third quartiles, respectively. The lines across the boxes indicate median values. C, controls; F, fibrosis. *P < 0.01; **P < 0.05.

**Figure 3:**
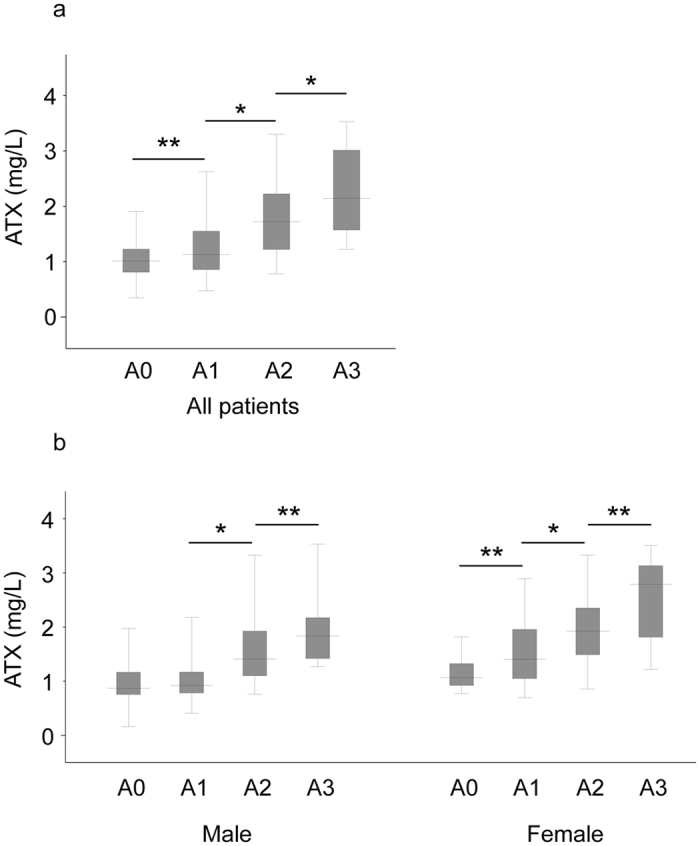
Correlation between serum autotaxin (ATX) and histological activity grade in all patients (**a**) and by gender (**b**). The top and bottom of each box represent the first and third quartiles, respectively. The lines across the boxes indicate median values. A, activity. *P < 0.01; **P < 0.05.

We excluded the 8 patients (6 male and 2 female) who were diagnosed as having F0 stage from the analysis to evaluate the association between ATX and histologically significant fibrosis. Correlations between ATX and fibrosis stage (Fig. 2) and activity grade (Fig. 3) of the liver were investigated for all subjects and according to gender. Overall, median ATX in healthy controls and patients with fibrosis stage F1, F2, F3, and F4 was 0.76, 1.05, 1.64, 1.93, and 2.16, respectively. Median ATX levels in male healthy controls and male patients with fibrosis stage F1, F2, F3, and F4 were 0.70, 0.90, 1.33, 1.56, and 2.17 mg/L, respectively, while those in women were 0.82, 1.33, 1.96, 2.21, and 2.16 mg/L, respectively. Serum ATX increased significantly according to liver fibrosis progression (*r* = 0.72, *P* < 0.001 overall*, r* = 0.77, *P* < 0.001 in men, and *r* = 0.73, and *P* < 0.001 in women) and activity (*r* = 0.46, *P* < 0.001 overall, *r* = 0.69, *P* < 0.001 in men, and *r* = 0.66, *P* < 0.001 in women). Female ATX levels at each fibrosis stage were significantly higher than corresponding values in men (F1: *P* < 0.001, F2: *P* < 0.001, F3: *P* < 0.005) apart from F4 (*P* = 0.708).

### Correlations between ATX and fibrosis markers

**Figure 4:**
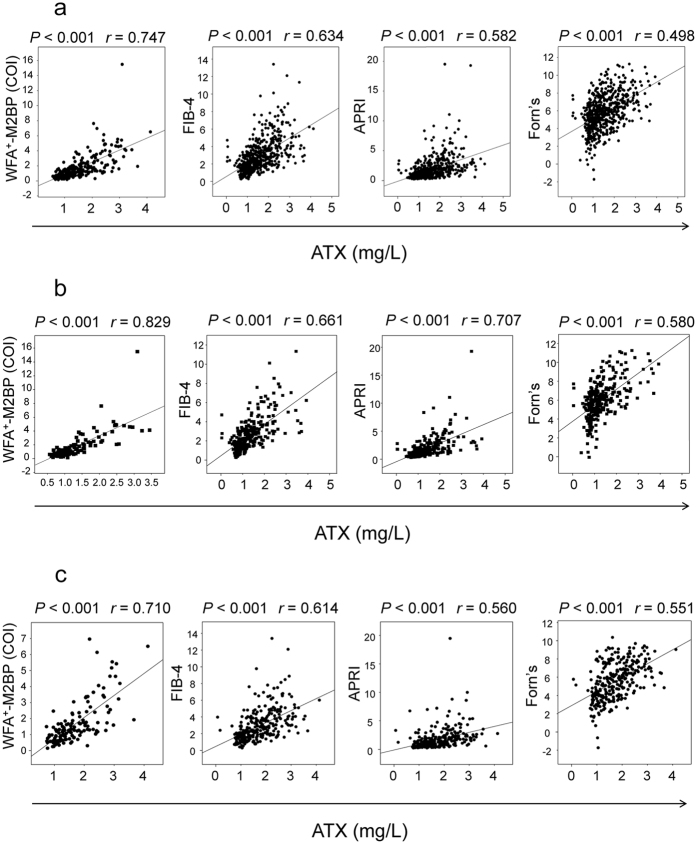
Correlation between serum autotaxin (ATX) and other fibrosis markers in total (**a**), male (**b**), and female (**c**) patients. WFA^+^-M2BP, *Wisteria floribunda* agglutinin-positive Mac-2-binding protein; FIB-4, FIB-4 index; APRI, aspartate aminotransferase-to-platelet ratio; Forn’s, Forn’s index.

The correlation coefficients between ATX and other clinical markers (albumin, AST, alanine aminotransferase (ALT), γ-glutamyltransferase (GGT), alpha-fetoprotein (AFP), and platelet count) and non-invasive fibrosis markers (WFA^+^-M2BP, APRI, FIB-4 index, and Forn’s index) for male and female patients after analysis by Spearman’s rank correlation coefficient test are shown in Table 2. ATX was significantly positively correlated to AST, ALT, GGT, and AFP and significantly negatively correlated to albumin and platelet count. We observed the strongest correlation between ATX and WFA^+^-M2BP in total (*r* = 0.75, *P* < 0.001), male (*r* = 0.83, *P* < 0.001), and female (*r* = 0.71, *P* < 0.001) patients in addition to remarkable correlations with other fibrosis markers (Fig. 4).

### Diagnostic performance of ATX

The calculated values of ATX for AUC, optimal cutoff value, sensitivity, specificity, positive predictive value, and negative predictive value for each fibrosis stage are listed in Table 3. The optimal cutoff values that best predicted fibrosis stage ≥F1 (vs. controls), ≥F2, ≥F3, and F4 were 0.9, 1.2, 1.5, and 1.7 mg/L, respectively, in all patients, 0.8, 1.1, 1.3, and 1.7 mg/L, respectively, in male patients, and 0.9, 1.7, 1.8, and 2.0 mg/L, respectively, in female patients.

### Comparison of fibrosis markers by AUC

**Figure 5:**
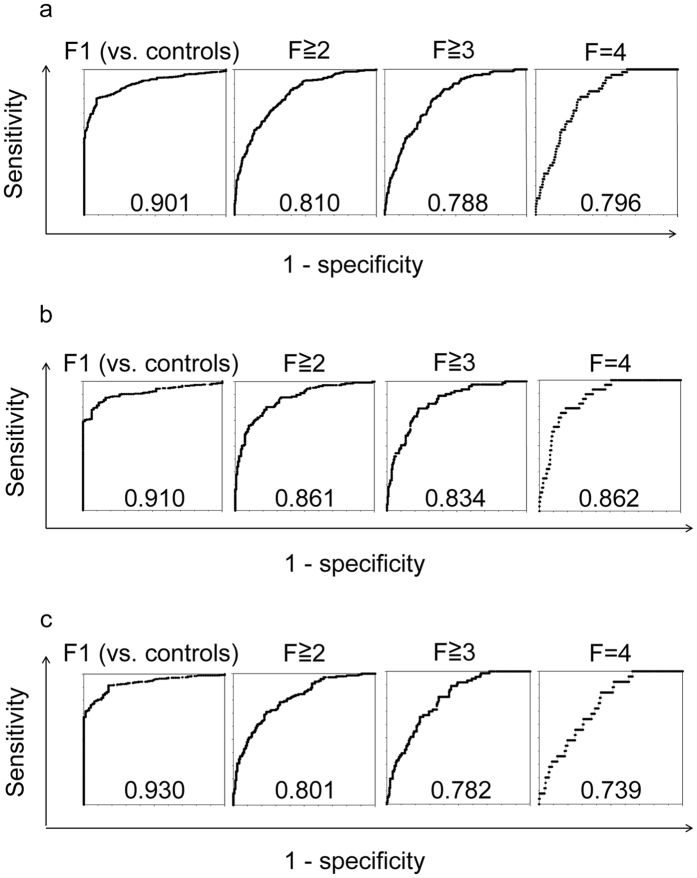
Receiver operating characteristic curves for autotaxin (ATX) for the estimation of early fibrosis (≥F1), significant fibrosis (≥F2), severe fibrosis (≥F3), and cirrhosis (F4) in total (**a**), male (**b**), and female (**c**) patients. The number at the bottom center of each graph is the area under the curve. F, fibrosis.

The ROC curves of ATX for predicting early fibrosis (≥F1 vs. controls), significant fibrosis (≥F2), severe fibrosis (≥F3), and cirrhosis (F4) in total, male, and female patients are presented in Fig. 5 and comparisons of AUC between ATX and other fibrosis markers in predicting fibrosis stage are summarized in Table 4. The AUC of ATX (0.901 overall, 0.910 in men, and 0.930 in women) for predicting early fibrosis (≥F1) was comparable to those of WFA^+^-M2BP and FIB-4 index. APRI was a significantly better predictor of early fibrosis than ATX for all subjects and both genders (0.982 [*P* < 0.01] overall, 0.985 [*P* < 0.01] in men, and 0.981 [*P* < 0.01] in women). The AUC of ATX (0.810 overall, 0.861 in men, and 0.801 in women) for predicting significant fibrosis (≥F2) was similar to those of WFA^+^-M2BP, FIB-4 index, APRI, and Forn’s index in women and superior to that of Forn’s index for all subjects (0.747 [*P* < 0.01]) and men (0.737 [*P* = 0.001]). The AUC of ATX (0.788 overall, 0.834 in men, and 0.782 in women) for prediction of severe fibrosis (≥F3) was comparable to those of WFA^+^-M2BP, FIB-4 index, APRI, and Forn’s index. For predicting cirrhosis, the AUC of ATX (0.796 overall, 0.862 in men, and 0.739 in women) closely reflected those of the other indicators.

## Discussion

This investigation confirmed that ATX levels were significantly higher in women than in men in both healthy controls and patients with HCV, as earlier reported^26^,^28^. Accordingly, we compared ATX values with those of clinical markers according to gender in the study, and would advocate the same approach for assessing liver fibrosis stage with this biomarker. Tokumura *et al*. found serum ATX activity to be increased in normal pregnant women^19^, possibly since it also stabilizes blood vessels and is essential for blood vessel formation during fetal development^29^,^30^. However, the precise reason for this discrepancy remains under debate. ATX may participate in female reproductive biology, which is an important issue that requires investigation in future studies comparing ATX by gender.

Serum levels of ATX were verified to correlate both with liver fibrosis stage as well as with other clinical and non-invasive fibrosis markers in our study cohort of 593 patients that was substantially larger than a previous report of 74 patients^27^. Notably, ATX levels were validated by biopsy-determined histological scores in all patients. During the progression of liver fibrosis, liver sinusoidal endothelial cells are known to exhibit a loss of several receptors and sinusoidal endothelial fenestrae, causing the capillarization of sinusoids and uptake impairment of various substances^31^. ATX is generally considered to be high in the serum despite being cleared from the circulation and degraded in liver sinusoidal endothelial cells within minutes^32^. The phenotypic changes occurring in the liver during liver fibrosis may lead to reduced ATX clearance, thereby increasing circulating ATX concentration. Pleli *et al*. reported that ATX levels correlated closely with Child-Pugh stage and model of end stage liver disease (MELD) score, suggesting that ATX was an indicator of liver injury severity^28^. Although we could not address their findings since patients with hepatic failure were excluded from our cohort, our data confirmed that ATX level was highest in cirrhosis (F4).

ROC assessment of ATX confirmed the enzyme to be a comparably reliable marker for detecting fibrosis stage, especially F1 and F2. DAAs are currently the standard of care in chronic HCV infection as these IFN-free regimens achieve high rates of a sustained virological response with few side effects. Patients who are elderly or who exhibit persistently normal ALT levels are particularly well suited for DAA therapy. It is becoming difficult to perform liver biopsy in such individuals due to refusal and contraindications that include anticoagulant and antiplatelet agents. Moreover, most patients with normal ALT levels have none to early fibrosis^33^. ATX may therefore be useful to accurately determine early to significant fibrosis stage prior to initiating DAA treatment in these patients.

Interestingly, ATX was positively correlated with ALT and AFP as well as with histological activity grade. Elevated ALT and AFP reflect liver inflammation and regeneration. A recent study showed that ATX might be causally linked to immune activation during HCV infection and uncovered a decline in ATX to levels comparable to those of uninfected participants within 24 weeks of DAA therapy^34^. ATX was also correlated with transaminase and APRI, which was compatible with our own data. Hence, serum ATX level may be associated not only with liver fibrosis, but also with liver inflammation and regeneration.

A longitudinal investigation of patients with HCV with respect to ATX and clinical features, including long-term prognosis and liver carcinogenesis, is needed. The clinical utility of ATX should be assessed in other chronic liver diseases as well. In addition, the latest EASL NAFLD guidelines have established alcohol consumption of 20 g ethanol daily for females and 30 g daily for males to exclude alcoholic liver disease^35^,^36^. We had originally set this limit at 60 g ethanol/day in 1982 when the first liver biopsies were performed and therefore only had patient data for ≤ or >60 g/day. Such differences in alcohol intake may have influenced fibrosis differently in chronic hepatitis C and added bias to the results of this study. Lastly, we cannot exclude the possibility that ATX levels may have been underestimated in patients with liver cirrhosis since liver biopsy is sometimes contraindicated in such individuals in the clinical setting.

In conclusion, ATX represents an accurate, non-invasive biomarker for liver fibrosis estimation in patients with chronic HCV infection and merits more comprehensive establishment based on gender.

## Methods

### Subjects

A total of 593 patients chronically infected with HCV, all of whom having received liver biopsy at Shinshu University Hospital in Matsumoto, Japan, between 1982 and 2015, were enrolled in this cross-sectional study. Their racial background was uniformly Japanese. The diagnosis of chronic hepatitis C was based on previously reported criteria as the presence of serum HCV antibodies and detectable HCV RNA^37^. One hundred and sixty subjects (80 male and 80 female) whose liver function tests were within normal levels were recruited as healthy controls. Age distribution was equally distributed among male and female controls (twenties: 20 subjects, thirties: 20 subjects, forties: 20 subjects, fifties: 20 subjects). All patients and controls were negative for hepatitis B surface antigen and antibodies to hepatitis B core antigen and the human immunodeficiency virus. No patients complicated with HCC were included. Patients who were diagnosed as having alcoholic liver disease, defined as an average daily consumption of >60 g of ethanol, were excluded. Patients who exhibited evidence of other liver disease, such as non-alcoholic liver disease, primary biliary cholangitis, or autoimmune hepatitis, were excluded as well. This study was reviewed and approved by the Institutional Review Board of Shinshu University Hospital (Matsumoto, Japan) (approval number: 3244), and written informed consent was obtained from all participating subjects. The investigation was conducted according to the principals of the Declaration of Helsinki.

### Laboratory testing

All laboratory data were obtained on the same day or within 14 days after liver biopsy. ALT, AST, GGT, AFP, and other relevant biochemical tests were performed using standard methods.

### Measurement of ATX

Blood samples were obtained on the same day or within 14 days after liver biopsy. Separated serum samples were immediately stored at −20 °C until testing.

Serum ATX antigen concentration was determined using a specific 2-site enzyme immunoassay. For preparation of the immunoassay, R10.23 was digested with pepsin, and the purified F(ab)_2_ form was isolated using phenyl-5PW hydrophobic column chromatography (Tosoh Co., Tokyo, Japan) to avoid the nonspecific binding of human antibodies to animal IgG types, such as human anti-mouse antibodies. In-house magnetic beads were coated with R10.23 F(ab)_2_ and placed in a reaction cup, and then 35 ng of alkaline phosphatase-labeled R10.21 in assay buffer (5% BSA, 5% sucrose, 10 mmol/l Tris–HCl, 10 mmol/l MgCl_2_, pH 7.4) was added. The ATX assay reagent was prepared by immediate freeze-drying of the reaction cup for subsequent use in a commercial automated immunoassay analyzer AIA-system (Tosoh Co.). The AIA-system process included automated specimen dispensation, incubation of the reaction cup, bound/free washing procedure, 4-methylumbelliferyl phosphate substrate dispensation, fluorometric detection, and result report. Antigen-antibody reaction time was 10 min and the first result was reported within 22 min. The throughput of the system was 60 samples/h using the AIA-600 II system^26^.

### Liver biopsy and histological evaluation

Liver biopsies were performed on all patients by percutaneous sampling of the right lobe with a 14-gauge ACECUT needle (TSK LABORATORY Co., Hyogo, Japan) or by laparoscopic liver biopsy with a 13-gauge Silverman needle (Olympus Co., Tokyo, Japan). All biopsy specimens were 1.5 cm or more in length. Formalin-fixed, paraffin-embedded specimens were prepared and used for histopathological studies. Sections of 4 μm in thickness were cut from each paraffin block and stained with hematoxylin and eosin, periodic acid-Schiff after diastase digestion, or Azan-Mallory staining. All liver biopsy samples were independently evaluated by 2 investigators (AM and ET) who were blinded to the clinical data. Fibrosis stage and activity grade were assessed independently on each histological section by both investigators. In the case of a discrepancy, histological sections were simultaneously reviewed using a multi-pipe microscope to reach a consensus. Liver fibrosis stage (F0-4) and activity grade (A0-3) were determined according to the METAVIR scoring system^38^, as follows: F0 = no fibrosis, F1 = portal fibrosis without septa, F2 = portal fibrosis with few septa, F3 = numerous septa without cirrhosis, and F4 = cirrhosis. Significant fibrosis was defined as ≥F2, severe fibrosis as ≥F3, and cirrhosis as F4.

### Other fibrosis markers

Serum WFA^+^-M2BP levels were measured simultaneously with ATX from stored serum samples obtained between 2006 and 2015. Serum WFA^+^-M2BP level was also measured in stored serum samples at the same point. Three additional surrogate blood indices of liver fibrosis were assessed at enrollment: FIB-4 index, APRI, and Forn’s index calculated according to the published formulae of (age [years] × AST [IU/L])/(platelet count [10^9^/L] × ALT [IU/L]^1^/^2^)^11^, (AST/upper limit of normal; 40 IU/L) × (100/platelet count [10^9^/L])^12^, and 7.811–3.131 × ln(platelet count [10^9^/L]) + 0.781 × ln(GGT [IU/L]) + 3.467 × log(age [years]) − 0.014 × (cholesterol [mg/dL])^13^, respectively.

### Statistical analysis

Statistical analysis and data visualization were carried out using IBM SPSS Statistics version 23.0 (IBM, Chicago, IL) and StatFlex version 6.0 (Artech Co., Ltd., Osaka, Japan) software. Data are presented as median ± interquartile range (IQR) for continuous variables. Groups were compared using the chi-square test for categorical variables. Correlations between fibrosis stage and serum ATX or other fibrosis markers were analyzed by means of Spearman’s rank test. Diagnostic accuracy was evaluated using the area under the receiver operating characteristic (ROC) curve (AUC). Comparisons of paired AUCs and 95% confidence intervals (CIs) were carried out using the nonparametric Delong test^39^. Cutoff values were identified by the Youden index, and the nearest clinically applicable value to the cutoff was considered as the optimal threshold for clinical convenience. All statistical tests were two-sided and evaluated at the 0.05 level of significance.

## Additional Information

**How to cite this article:** Yamazaki, T. *et al*. Association of Serum Autotaxin Levels with Liver Fibrosis in Patients with Chronic Hepatitis C. *Sci. Rep.* **7**, 46705; doi: 10.1038/srep46705 (2017).

**Publisher's note:** Springer Nature remains neutral with regard to jurisdictional claims in published maps and institutional affiliations.

## Figures and Tables

**Table 1:** Baseline clinical characteristics of 593 patients with chronic hepatitis C.

	All patients (n = 593)		Male (n = 292)		Female (n = 301)		*P* value*
	Median	IQR	Median	IQR	Median	IQR	
Age at entry (years)	58	(51–64)	56	(48–62)	60	(53–66)	<0.001
Laboratory data							
AST (U/L)	48	(33–85)	49	(34–82)	47	(32–87)	0.422
ALT (U/L)	64	(37–111)	68	(43–122)	57	(34–107)	0.002
GGT (U/L)	38	(21–72)	52	(27–95)	29	(20–49)	<0.001
T-Bil (mg/dL)	0.80	(0.62–1.00)	0.80	(0.61–1.01)	0.78	(0.62–1.00)	0.444
AFP (ng/mL)	4.5	(2.9–9.5)	4.2	(2.7–9.6)	4.8	(3.0–9.4)	0.491
Fibrosis markers							
ATX (mg/L)	1.39	(1.01–1.99)	1.16	(0.85–1.68)	1.64	(1.19–2.20)	<0.001
WFA^+^-M2BP^a^	1.25	(0.77–2.12)	1.12	(0.74–2.19)	1.29	(0.85–1.92)	0.549
FIB-4 index	2.31	(1.57–3.60)	2.21	(1.51–3.28)	2.39	(1.62–3.86)	0.037
APRI	1.14	(0.66–2.11)	1.12	(0.71–2.07)	1.14	(0.63–2.12)	0.440
Forn’s index	5.82	(4.42–7.06)	5.98	(4.90–7.11)	5.55	(4.01–7.01)	0.011
Histological findings							
Fibrosis stage (0/1/2/3/4)	8/284/144/103/54		6/137/67/54/28		2/147/77/49/26		0.888
Activity grade (0/1/2/3)	47/229/262/26		30/107/123/12		17/122/139/14		0.171

Abbreviations: IQR, interquartile range; AST, aspartate aminotransferase; ALT, alanine aminotransferase; GGT, γ-glutamyltransferase; AFP, alpha-fetoprotein; ATX, autotaxin; WFA^+^-M2BP, *Wisteria floribunda* agglutinin-positive Mac-2-binding protein; APRI, AST-to-platelet ratio.^a^ WFA^+^-M2BP was measured in 237 samples obtained between 2006 and 2015. *Comparison between male and female subjects.

**Table 2:** Correlation coefficients between ATX and other clinical and fibrosis markers.

		AST	ALT	GGT	AFP	PLT	WFA^+^-M2BP	FIB-4 index	APRI	Forn’s index
All patients	*r*	0.488	0.329	0.343	0.561	−0.483	0.747	0.634	0.582	0.498
	*P*	<0.001	<0.001	<0.001	<0.001	<0.001	<0.001	<0.001	<0.001	<0.001
Male	*r*	0.601	0.444	0.441	0.641	−0.564	0.829	0.661	0.707	0.580
	*P*	<0.001	<0.001	<0.001	<0.001	<0.001	<0.001	<0.001	<0.001	<0.001
Female	*r*	0.469	0.376	0.557	0.547	−0.468	0.710	0.614	0.560	0.551
	*P*	<0.001	<0.001	<0.001	<0.001	<0.001	<0.001	<0.001	<0.001	<0.001

Abbreviations: ATX, autotaxin; AST, aspartate aminotransferase; ALT, alanine aminotransferase; GGT, γ-glutamyltransferase; AFP, alpha-fetoprotein; PLT, platelet count; WFA^+^-M2BP, Wisteria floribunda agglutinin-positive Mac-2-binding protein; APRI, AST-to-platelet ratio.

Data were analyzed by Spearman’s rank correlation coefficient test for each gender.

**Table 3:** Diagnostic performance of ATX in all, male, and female patients.

Fibrosis stage	Cutoff	AUC	(95% CI)	Sensitivity (%)	Specificity (%)	PPV (%)	NPV (%)
All patients							
≥F1 (vs. controls)	0.9	0.901	(0.879–0.923)	82	85	95	56
≥F2	1.2	0.810	(0.775–0.845)	83	62	69	78
≥F3	1.5	0.788	(0.749–0.828)	79	68	46	89
=F4	1.7	0.796	(0.745–0.847)	79	69	20	97
Male							
≥F1 (vs. controls)	0.8	0.910	0.881–0.940	83	88	96	60
≥F2	1.1	0.861	0.819–0.903	81	71	74	78
≥F3	1.3	0.834	0.783–0.884	79	74	54	90
=F4	1.7	0.862	0.803–0.921	75	81	30	97
Female							
≥F1 (vs. controls)	0.9	0.930	0.905–0.956	92	75	93	72
≥F2	1.7	0.801	0.752–0.851	71	77	76	72
≥F3	1.8	0.782	0.726–0.837	71	65	40	87
=F4	2.0	0.739	0.654–0.823	64	68	16	95

Abbreviations: ATX, autotaxin; AUC, area under the curve; CI, confidence interval; PPV, positive predictive value; NPV, negative predictive value; F, fibrosis.

**Table 4:** Comparisons of AUC between ATX and other fibrosis markers in predicting fibrosis stage for all, male, and female patients.

	ATX	WFA^+^-M2BP	FIB-4 index	APRI	Forn’s index
All patients					
≥F1 (vs. controls)	0.901	0.916	0.926	0.982^a^	ND
≥F2	0.810	0.812	0.783	0.788	0.747^b^
≥F3	0.788	0.776	0.814	0.780	0.770
=F4	0.796	0.814	0.859	0.808	0.797
Male					
≥F1 (vs. controls)	0.910	0.925	0.913	0.985^a^	ND
≥F2	0.861	0.829	0.777^b^	0.801	0.737^a^
≥F3	0.834	0.794	0.808	0.779	0.758
=F4	0.862	0.824	0.892	0.842	0.823
Female					
≥F1 (vs. controls)	0.930	0.907	0.938	0.981^a^	ND
≥F2	0.801	0.794	0.787	0.768	0.761
≥F3	0.782	0.750	0.822	0.772	0.780
=F4	0.739	0.807	0.825	0.769	0.769

Abbreviations: AUC, area under the curve; ATX, autotoxin; WFA^+^-M2BP, Wisteria floribunda agglutinin-positive Mac-2-binding protein; APRI, AST-to-platelet ratio; ND, not determined; F, fibrosis. ^a^P < 0.01 vs. ATX; ^b^P < 0.05 vs. ATX.
